# Ranking Methodology for Evaluating Region-Wise COVID-19 Testing Performance in India

**DOI:** 10.3389/fpubh.2022.887665

**Published:** 2022-07-19

**Authors:** Jasmine Kaur, Jasleen Kaur, Harpreet Singh

**Affiliations:** Division of Biomedical Informatics, Indian Council of Medical Research, New Delhi, India

**Keywords:** COVID-19, ranking methodology, testing performance, COVID-19 testing performance, region-wise COVID-19 testing

## Abstract

For containment of COVID-19, most countries are following the isolate, test, treat and trace approach. Following the approach, India scaled up COVID-19 testing from about 5,000 tests per day at the end of March 2020 to more than 1 M tests per day in September 2020. Testing scale-up has seen a huge variation across states and union territories (UTs) with respect to growth rates, testing strategies, testing infrastructure, and deployment of various kit types (RT-PCR, Antigen, CBNAAT, etc). To understand the gaps in testing and prioritize appropriate interventions, it is important for national stakeholders to evaluate and rank states/UTs based on their testing performance. Indicators like total samples tested, total samples positive, tests per million, and positives per million are currently being used by epidemiologists and researchers for comparing the performance of various regions. This article proposes a robust ranking methodology to rank the states/UTs in India based on a comprehensive scoring developed by combining multiple variables for evaluating the testing performance of states/UTs. Since RT-PCR testing is considered the gold standard for COVID-19 testing, the composite score for testing performance in this article is defined by the ability of states/UTs to successfully deploy RT-PCR testing and reduce its positivity over time. Evaluating region-wise ranking can enable the identification of areas requiring immediate attention in poorly performing regions, thus channelizing efforts and resources in the right direction.

## Introduction

The novel coronavirus COVID-19 originated in China in December 2019. It rapidly spread across the globe, eventually becoming a pandemic in March 2020 (1). WHO recommended governments isolate, test, and treat every case to quell and control the pandemic effectively (2). India detected its first case of COVID-19 on 30th Jan 2020 (3). Following the same approach, India augmented its testing capacity more than 200 times and is testing more than 1 million samples per day (4). Augmenting the capacity and scaling up of COVID-19 testing has played a crucial role in India's response to the pandemic. Despite huge disparities across states and union territories (UTs), this scale-up has been achieved in terms of testing volumes, infrastructure, product mix, strategies, and timelines. Going forward, states/UTs in India need to continue the scale-up in the positive direction to keep the pandemic in check. To accomplish this, the national stakeholders in India must continuously evaluate the state/UT testing performance in order to identify areas for development and achieve optimal testing performance.

The COVID-19 testing varies across states and UTs with respect to growth rates, testing strategies, testing infrastructure, and deployment of various kit types (RT-PCR, Antigen, CBNAAT, etc). Different countries, such as India (5) and USA (6, 7), among others, have ranked their states/UTs based upon the total samples tested and total samples positives (5–8). Indicators like total samples tested, total samples positive, tests per million, and positives per million are currently being used by epidemiologists and researchers for comparing the performance of various regions. For example, to measure COVID-19 testing performance, commonly used indicators include tests per million population, positivity rate, and rate of change of these indicators. A report from Niti Aayog (9) in 2019 describes creating a composite health index wherein scaling of indicators was done to compute a final composite score. For COVID-19, no composite indicators have been reported at a national level to date. This article aims to describe a comprehensive ranking methodology for evaluating the testing performance of states/UTs in India.

## Methodology

### Data Sources

1. **Testing and positivity data**: The total COVID-19 tests and positives detected in each state bifurcated by kit type (RT-PCR, Antigen, and Overall) for five consecutive weeks: 22–28 October 2020, 29 October−5 November 2020, 6–12 November 2020, 13–19 November 2020, and 20–26 November 2020. This aggregated data is taken from the ICMR's COVID-19 database (4) containing a line list of all COVID-19 tests conducted in the country.
2. **State population:** State-wise projected population as of 31st May 2020 by Unique Identification Authority of India (10).

### Variables for Ranking

The following variables have been calculated at a state level for the scoring process:

1. **% RT-PCR tests** in the selected week are defined as:

$$\begin{array}{l} \%\;RT\;PCR\;tests=\frac{Total\;samples\;tested\;using\;RT\;PCR}{Total\;samples\;tested\;}\;*100 \end{array}$$

2. **RT-PCR positivity (%)** in the selected week is defined as:

$$\begin{array}{l} \%\;RT\;PCR\;positivity=\frac{Total\;positives\;detected\;using\;RT\;PCR}{Total\;samples\;tested\;using\;RT\;PCR}\\ \;*100 \end{array}$$

3. **RT-PCR tests per million (TPM)** in the selected week are defined as:

$$\begin{array}{l} RT\;PCR\;tests\;per\;million\;(TPM)\\ \;=\frac{Total\;samples\;tested\;using\;RT\;PCR}{Population\;of\;the\;state}\;*\;1,000,000 \end{array}$$

4. **Change in RT PCR positivity (%)** is defined as:

$$\begin{array}{l} \%\;Change\;in\;RT\;PCR\;positivity\\ =\frac{\text{RTPCRpositivity}(\%)(\text{selectedweek-previousweek})}{\text{RT PCR positivity }(\%)\text{ previous week}}*100 \end{array}$$

5. **Change in RT PCR TPM (%)** is defined as:

$$\begin{array}{l} \%\;Change\;in\;RT\;PCR\;TPM\\ \;=\frac{\text{RT PCR TPM }(\%)\;(\text{selected week - previous week})}{\text{RT PCR TPM }(\%)\text{ previous week}}*100 \end{array}$$

### Ranking Methodology

The ICMR data analysis portal (4) has been used to collect state-level numbers for each of the five variables stated above, and the standardized scores at the state level for each of these variables have been calculated using the formula below.


$$\begin{array}{l} Z=\frac{x-\mu }{\sigma } \end{array}$$

Where *Z* = standardized score, *x* = observed value for the variable and state, μ = mean of the variable, and σ = standard deviation of the variable.

For variables RT-PCR positivity (%) in the last week and change in RT-PCR positivity (%), a higher *Z*-value reflects a poor score for the two variables i.e., RT-PCR positivity (%) in the last week and change in RT-PCR positivity (%), whereas a higher value reflects a higher score for the other three variables i.e., % RT-PCR tests, RT-PCR tests per million (TPM), and change in RT PCR TPM (%). Therefore, the variables RT-PCR positivity (%) in the last week and change in RT-PCR positivity (%) have been multiplied by −1 to get the final standardized scores, which reflect the fact that higher scores imply higher ranking across the five variables.

After calculating standardized scores, each of the standardized variables has been given equal weights (20%), and a composite score for each state has been calculated by adding the sum of the standardized scores and the weights of the five variables.


$$\begin{array}{l} Composite\;score=\sum \;\left(\text{Weight }*Standardized\;score\right) \end{array}$$

Before assigning equal weights to all the five variables, a correlation matrix has been made for standardized scores of all variables for all last 5 weeks. Ideally, variables with high correlation (Pearson correlation coefficient > 0.7) should not be assigned the same weights (11) in creating the composite score since the combination of highly correlated variables can skew the composite score. For correlated variables, different methods as described by Becker et al. can be used (12) for decomposition that minimizes the effect caused by correlations of variables for assigning the weights based on the dataset. The correlation matrix for all the 5 weeks showed that none of the five variables were substantially correlated with each other, therefore equal weighting was justified.

## Results

For the practical demonstration, the state-wise ranking of testing performed for the week 20^th^-26th November 2020 is shown in Table 1. The top five states/UTs in order of testing performance were S1, S2, S3, S4, and S5 (as shown in Table 1), according to composite scores obtained for each state for the selected week: 20th−26th November 2020. The top five states/UTs have a high percent of RT-PCR tests, low RT-PCR positivity, high RT-PCR tests per million, declining RT-PCR positivity (percent), and increasing RT-PCR tests per million, as seen in Table 1. The bottom five states, on the other hand, exhibit reverse behavior for most of the variables and require actions to enhance testing metrics where they lag the others.

**Table 1:** State-wise ranking of Indian states on testing performance by the composite index for the week 20–26 November 2020.

**STATE**	**Rank**	% RT-PCR tests last week*	RT-PCR positivity (%) last week*	RT-PCR tests per million (TPM) last week*	Change in RT-PCR positivity (%)*	Change in RT-PCR TPM (%)*
S1	1	89%	0%	2,956	−90%	104%
S2	2	84%	1%	10,227	−17%	23%
S3	3	98%	2%	5,637	−3%	9%
S4	4	57%	8%	2,740	−37%	114%
S5	5	80%	4%	4,175	1%	34%
S6	6	72%	4%	4,905	−2%	31%
S7	7	75%	12%	5,563	−16%	21%
S8	8	40%	2%	3,974	−30%	29%
S9	9	43%	22%	8,862	−23%	42%
S10	10	98%	19%	1,858	−22%	51%
S11	11	86%	17%	1,799	−14%	64%
S12	12	43%	4%	2,235	−13%	47%
S13	13	21%	10%	1,141	−26%	113%
S14	14	31%	1%	955	−26%	43%
S15	15	56%	11%	2,503	−20%	35%
S16	16	25%	13%	1,689	−6%	95%
S17	17	40%	5%	1,416	−12%	23%
S18	18	45%	10%	1,091	−3%	52%
S19	19	4%	4%	76	−65%	19%
S20	20	37%	15%	2,879	−28%	15%
S21	21	32%	3%	2,101	−15%	−15%
S22	22	30%	11%	1,924	−15%	31%
S23	23	10%	2%	631	−16%	39%
S24	24	19%	8%	1,446	−18%	33%
S25	25	35%	2%	1,101	−3%	−4%
S26	26	13%	6%	842	−19%	7%
S27	27	55%	17%	1,962	2%	−12%
S28	28	40%	15%	1,195	−1%	5%
S29	29	23%	17%	1,420	24%	69%
S30	30	43%	30%	4,906	−12%	−11%
S31	31	17%	2%	544	13%	−9%
S32	32	50%	0%	41	0%	−77%
S33	33	8%	1%	1,508	12%	−29%
S34	34	17%	7%	1,110	−5%	−31%
S35	35	12%	3%	649	13%	−16%
S36	36	48%	31%	517	144%	−28%

* *Color gradient from red to green represents poor to good*.

However, if we compare consecutive 4-week composite indexes for all the states, 19 states increased their ranking from the week of 20–6 November 2020 to the week of 29 October−5 November 2020. The states have moved up in the rankings owing to improvement in testing numbers and vice versa. The state S4, for example, was ranked 31 in the week of October 29th to November 5th, despite being ranked 9 in the week of November 20th to 26th. The testing metrics have been improved in the multiple ways: (a) increase in % RT PCR tests from 27 to 43%; (b) decrease in RT PCR positivity from 26 to 22%; (c) increase in RT PCR TPM from 5,976 to 8,862; (d) week over week % change in RT PCR positivity from +44 to −23%; (e) week over week % change in RT PCR TPM from +9 to +42%. Because of a high percent of RT-PCR tests (>98%), low positive (3.2%), high tests per million (2–2.5 times the national average), and decreasing positivity week after week, the state S3 has been ranked third for the last 4 weeks. Likewise, in the last 4 weeks, State S35 has regularly been in the bottom half of the rankings, owing to a low percentage of RT-PCR tests (11–14%) and low tests per million (26–38% of the national average), with tests per million decreasing week after week.

## Discussion and Conclusion

This article describes a composite index that will be calculated considering the testing done in the previous week. The states/UTs can be ranked based on the composite index. Further, once an intervention is implemented by the states, a change in the ranking of the states in the consecutive weeks can help the stakeholders in analyzing the effect of their interventions. Thus, the ranking methodology discussed in this article can be used to evaluate and rank states/UTs/districts based on their testing performance across the several metrics and provide a directional sense of the gaps that would require focused efforts to improve the testing outcomes. For e.g., the States/UTs with an RT-PCR TPM that is less than the national average and RT-PCR positivity greater than the national average should be prime targets for improving RT-PCR coverage. This methodology can also be used to compare the performance of regions or facilities on key programmatic indicators in other national and state programs. The publication of these rankings on a regular basis can promote healthy competition among states/UTs to improve their performance across critical indicators, which will enable them to attain the optimum programmatic outcomes. A limitation of this methodology is that it just aims to rank the states and UTs based on their current testing performance and does not take into account the qualitative factors, such as governance, IEC activities, population demographics, and social behavior, which are also important in determining testing outcomes. In future work, the ranking of the states/UTs using this proposed ranking methodology can be used for optimizing the testing performance in poorly performing & low resource regions by integrating a model and channelizing efforts & resources in the right direction (13).

## Data Availability Statement

The original contributions presented in the study are included in the article/supplementary material, further inquiries can be directed to the corresponding author/s.

## Ethics Statement

The study has been approved by the Director-General, Indian Council of Medical Research. The study does not require taking informed consent from all patients as no patient is sampled exclusively for the study.

## Author contributions

HS and JasmK have conceptualized the work and devised the methodology. JasmK and JaslK have written the manuscript. HS has critically reviewed the manuscript. All authors listed have made a substantial, direct, and intellectual contribution to the work and approved it for publication.

## Funding

This study has been funded by the Indian Council of Medical Research, New Delhi (No- ISRM/14(01)/TF/Genom/2017).

## Conflict of Interest

The authors declare that the research was conducted in the absence of any commercial or financial relationships that could be construed as a potential conflict of interest.

## Publisher's note

All claims expressed in this article are solely those of the authors and do not necessarily represent those of their affiliated organizations, or those of the publisher, the editors and the reviewers. Any product that may be evaluated in this article, or claim that may be made by its manufacturer, is not guaranteed or endorsed by the publisher.
